# Predictive model for PSA persistence after radical prostatectomy using machine learning algorithms

**DOI:** 10.3389/fonc.2024.1452265

**Published:** 2024-12-06

**Authors:** Haotian Du, Guipeng Wang, Yongchao Yan, Shengxian Li, Xuecheng Yang

**Affiliations:** Department of Urology, The Affiliated Hospital of Qingdao University, Qingdao, China

**Keywords:** radical prostatectomy, PSA persistence, machine learning, random forest algorithm, prediction model

## Abstract

**Objective:**

To evaluate the efficacy of a machine learning model for predicting prostate-specific antigen (PSA) persistence after radical prostatectomy (RP).

**Methods:**

Data from 470 patients who underwent RP at the Affiliated Hospital of Qingdao University from January 2018 to June 2021 were retrospectively analyzed. Ten risk factors, including age, body mass index (BMI), preoperative PSA, biopsy Gleason score, total prostate specific antigen density (PSAD), clinical tumor stage, clinical lymph node status, seminal vesicle invasion, capsular invasion and positive surgical margin, were included in the analysis. The data were randomly divided into a training set and a test set at a ratio of 7:3, and seven different machine learning algorithms were compared. The confusion matrix, receiver operating characteristic (ROC) curve and area under the ROC curve (AUC) were used to evaluate the diagnostic performance of the model, and the random forest algorithm found to be the optimal prediction model.

**Results:**

In the entire cohort, 142 (30.21%) patients developed PSA persistence. Based on all included risk factors, the random forest model had the best effect among the seven models, with an AUC of 0.8607 in the training set and 0.8011 in the test set. The feature importance results showed that capsular invasion, positive surgical margin, preoperative PSA and biopsy Gleason score were the four most important risk factors for PSA persistence after RP.

**Conclusion:**

The Random Forest algorithm performed excellently in this study and can be used to construct a predictive model for PSA persistence. By incorporating clinical data from the Asian region and exploring the risk factors for PSA persistence, this study contributes to the existing research and aids clinicians in assessing the risk of PSA persistence occurrence, enabling timely treatment planning and improving patient prognosis.

## Introduction

Prostate cancer is the second most frequently diagnosed malignancy among males worldwide (1). In the United States and Europe, prostate cancer accounts for 29% to 23.2% of newly diagnosed cancers and 11% to 10.3% of cancer-related deaths (2, 3). In China, its incidence and mortality rates are increasing significantly, leading to a more urgent need for prevention and control strategies (4).

As prostate cancer becomes more common worldwide, more research has examined its treatment. Among a variety of options, radical prostatectomy (RP) remains one of the most effective treatments for localized and locally advanced prostate cancer (5, 6). However, treatment plans should be developed promptly in the event of surgical failure, with the aim of minimizing the risk of adverse impacts on the patient’s physical well-being and financial circumstances. Therefore, it is imperative to exercise prudence in decision-making regarding the management of prostate cancer and to conduct comprehensive preoperative and postoperative assessments. Prostate-specific antigen (PSA) is commonly measured in the follow-up of patients after RP. PSA is thought to be undetectable (< 0.1 ng/mL) after RP, and persistent PSA (≥ 0.1 ng/mL) is considered a failure of curative treatment. Persistent PSA is associated with worse oncologic outcomes after RP (7, 8). The latest guidelines for prostate cancer treatment also propose that persistent PSA should be regarded as a crucial parameter for assessing RP (9). Therefore, the establishment of an accurate prediction model for PSA persistence is highly important for evaluating the efficacy of RP and guiding treatment decisions.

Machine learning is a data-driven application of artificial intelligence. It can be used to autonomously exploit datasets to identify several variables and complex relationships between them. In recent years, machine learning techniques have been widely applied in modern molecular studies to construct predictive models (10). Machine learning techniques have also been employed in prostate cancer research, including models for the prediction of disease progression and specific mortality (11, 12). Machine learning encompasses various algorithms, among which random forest stands as a prominent one. Random forest is a learning method based on the construction of multiple classification trees. The main advantages of the proposed method are its robustness against overfitting and its user friendliness (13). Therefore, we constructed models to predict PSA persistence after RP using a random forest model and discussed the importance of each factor. We also discussed the guiding implications of this predictive model for clinical practice.

## Patients and methods

### Data collection

Clinical data were collected from 632 patients with prostate cancer who underwent radical prostatectomy at our center between January 2018 and June 2021. All patients underwent laparoscopic radical prostatectomy (LRP) or robot-assisted laparoscopic radical prostatectomy (RALP) in our hospital. The surgeries were performed by clinical physicians with over five years of surgical experience who have passed the surgical learning curve. The surgical approach was either extraperitoneal or transperitoneal. According to the European and Chinese guidelines for prostate cancer treatment, there are no significant differences in oncological and functional outcomes between LRP, RALP, and open surgery. Whether lymph node dissection is performed follows the standards of the Chinese Clinical Guidelines for Prostate Cancer, which recommend ePLND for intermediate- to high-risk prostate cancer with a lymph node-positive risk greater than 5% as assessed by the Briganti nomogram. All specimens were reviewed by pathologists from our hospital’s pathology department, and the pathology reports were audited by another senior pathologist with over 7 years of clinical experience.

We defined PSA persistence as a PSA concentration ≥0.1 ng/ml at 6-8 weeks after RP. Patients were stratified according to persistent PSA (PSA ≥0.1 ng/ml at 6-8 weeks after RP) versus undetectable PSA (PSA<0.1 ng/ml).

The inclusion criteria were as follows: (1) no history of neoadjuvant or adjuvant androgen deprivation treatment; (2) had a PSA examination within 6-8 weeks after RP; and (3) had detailed clinical and pathological data. Exclusion criteria were as follows:(1) Patients with positive postoperative pathological lymph nodes, since such patients need to undergo immediate endocrine therapy;(2) patients at high risk level without lymph node cleaning during RP; After applying these criteria, a total of 470 patients were included for analysis.

### Covariates

The covariates included age, body mass index (BMI), preoperative PSA, biopsy Gleason score, total prostate specific antigen density (PSAD), clinical tumor stage, clinical lymph node status, seminal vesicle invasion, capsular invasion, and positive surgical margin. The indicators mentioned above were selected based on previous studies on the analysis of PSA persistence (8, 14). To meet the requirements of different machine learning algorithms, we converted continuous variables into discrete variables, as detailed in **Table 1**. Categorical variables are presented as frequencies and percentages.

**Table 1:** Collation of the risk factors.

Factor	Value	PSA persistence, N (%)	No PSA persistence, N (%)
Age	0(≤50)	5(1.06%)	1(0.21%)
	1(50-60)	16(3.40%)	38(8.08%)
	2(60-70)	70(14.89%)	175(37.23%)
	3(>70)	51(10.85%)	114(24.25%)
BMI	0(<18.5 kg/m^2^)	6(1.28%)	5(1.06%)
	1(18.5-24.9 kg/m^2^)	56(11.91%)	102(21.70%)
	2(24-28 kg/m^2^)	52(11.06%)	166(35.32%)
	3(≥28 kg/m^2^)	28(5.96%)	55(11.70%)
Preoperative PSA	0(<10 ng/ml)	20(4.26%)	119(25.32%)
	1(10-20 ng/ml)	30(6.38%)	93(19.78%)
	2(>20ng/ml)	93(19.78%)	115(24.47%)
Biopsy Gleason score	0(<7)	4(0.85%)	54(11.49%)
	1(=7)	25(5.32%)	131(27.87%)
	2(>7)	113(24.04%)	143(30.43%)
PSAD	0(≤0.15)	39(8.30%)	140(29.79%)
	1(>0.15)	103(21.91%)	188(40.00%)
Clinical tumor stage	T1	14(2.98%)	110(23.40%)
	T2	62(13.19%)	202(42.98%)
	T3	54(11.49%)	12(2.55%)
	T4	12(2.55%)	4(0.85%)
Clinical lymph node status	0(lymph node negative)	114(24.26%)	289(61.49%)
	1(lymph node positive)	28(5.96%)	39(8.30%)
Seminal vesicle invasion	0(negative)	87(18.51%)	317(67.44%)
	1(positive)	55(11.70%)	11(2.34%)
Capsular invasion	0(negative)	77(16.38%)	312(66.38%)
	1(positive)	65(13.83%)	16(3.40%)
Positive surgical margin	0(negative)	41(8.72%)	225(47.87%)
	1(positive)	101(21.49%)	103(21.91%)

### Statistical analyses

The data were randomly divided into a training set and a test set at a ratio of 7:3, and seven typical machine learning algorithms, including logistic regression, support vector machine, LightGBM, random forest, extreme gradient boosting (XGBoost), Gaussian naive Bayes and k-nearest neighbor algorithms, were run via Python 3.9 to analyze the data. The confusion matrix, receiver operating characteristic (ROC) curve and area under the ROC curve (AUC) were used to evaluate the diagnostic performance of the model. The Random Forest algorithm was found to be the optimal model for prediction. After selecting the optimal model, the feature importance of each factor was calculated.

## Results

We identified and analyzed 470 patients who underwent RP at our center between January 2018 and June 2021 (**Table 1**), including 142 patients (30.21%) who experienced PSA persistence.

**Figure 1:**
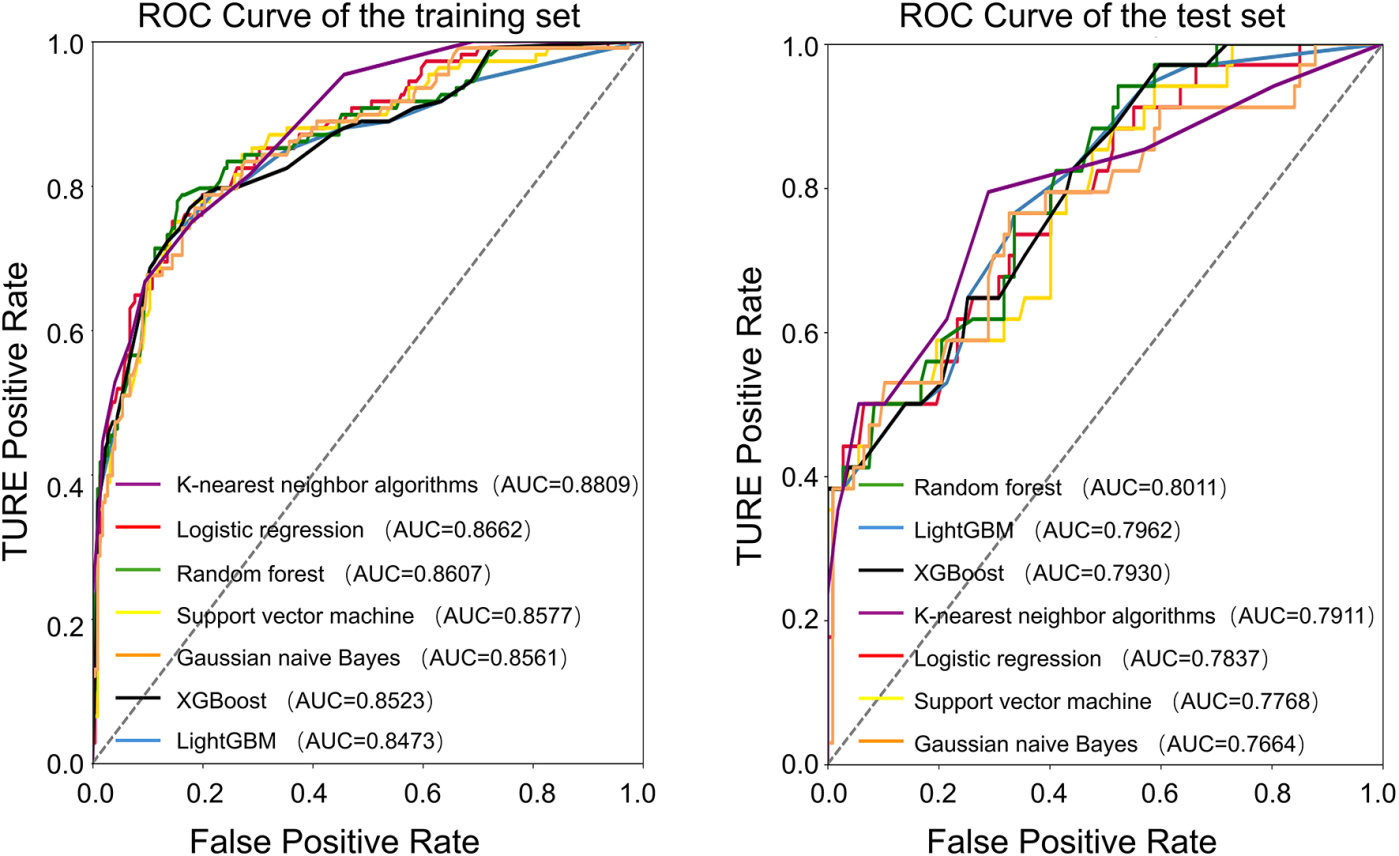
ROC curve of the training and test set.

The average age of the patients was 69 years. The patients were randomly divided into a training set and a test set at a ratio of 7:3. All factors in **Table 1** were included, and seven machine learning algorithms were run via Python 3.9. The obtained results showed the accuracy and AUC of the seven machine learning models in the training set and test set (**Tables 2**, **3**), and the respective ROC curves were constructed according to the data model validation set (**Figure 1**). The random forest model had the highest AUC of 0.8011(95%CI:0.7143,0.8710). The confusion matrices of the random forest model and the other six models were also made (**Table 2**). The number of correct predictive values included 14 cases of PSA persistence and 101 cases of undetectable PSA. The number of false predictive values included 6 cases of PSA persistence and 20 cases of undetectable PSA. The overall accuracy of the confusion matrix was 0.8156 and the precision was 0.7000. It is noteworthy that although the accuracy of the random forest model is not the best, considering the class imbalance in the dataset of this study, this is consistent with the real-life scenario where the number of patients with PSA persistence is significantly fewer than those without PSA persistence. Hence, the ability of the model to effectively reflect its classification capability on an imbalanced dataset, as indicated by the AUC, is more important to us. We believe that the random forest model, which has the highest and only AUC exceeding 0.8, exhibits the best performance.

**Table 2:** Confusion matrices for other six machine learning models.

Machine learning models		Training set			Test set	
		Predicted class			Predicted class	
	Actual class			Actual class		
Logistic regression		No PSA persistence	PSA persistence		No PSA persistence	PSA persistence
	No PSA persistence	206	15	No PSA persistence	92	15
	PSA persistence	41	67	PSA persistence	17	17
Support vector machine		No PSA persistence	PSA persistence		No PSA persistence	PSA persistence
	No PSA persistence	212	9	No PSA persistence	99	8
	PSA persistence	41	67	PSA persistence	17	17
LightGBM		No PSA persistence	PSA persistence		No PSA persistence	PSA persistence
	No PSA persistence	211	10	No PSA persistence	101	6
	PSA persistence	56	52	PSA persistence	20	14
XGBoost		No PSA persistence	PSA persistence		No PSA persistence	PSA persistence
	No PSA persistence	218	3	No PSA persistence	107	0
	PSA persistence	68	40	PSA persistence	21	13
Gaussian naive Bayes		No PSA persistence	PSA persistence		No PSA persistence	PSA persistence
	No PSA persistence	206	15	No PSA persistence	101	6
	PSA persistence	53	55	PSA persistence	20	14
K-nearest neighbor algorithms		No PSA persistence	PSA persistence		No PSA persistence	PSA persistence
	No PSA persistence	206	15	No PSA persistence	101	6
	PSA persistence	45	63	PSA persistence	21	13
Random Forest		No PSA persistence	PSA persistence		No PSA persistence	PSA persistence
	No PSA persistence	211	10	No PSA persistence	101	6
	PSA persistence	57	51	PSA persistence	20	14

**Table 3:** Accuracy and AUC of the seven machine learning models in the training and test sets.

machine learning models	Accuracy of the training set	Accuracy of the test set	AUC of the training set	AUC of the test set(95%CI)
Logistic regression	0.8298	0.7730	0.8662	0.7837(0.6866,0.8607)
Support vector machine	0.8480	0.8226	0.8577	0.7768(0.6692,0.8557)
LightGBM	0.7994	0.8156	0.8473	0.7962(0.6311,0.8257)
Random forest	0.7964	0.8156	0.8607	0.8011(0.7143,0.8710)
XGBoost	0.7842	0.8511	0.8523	0.7930(0.6931,0.8664)
Gaussian naive Bayes	0.7933	0.8156	0.8561	0.7664(0.6746,0.8519)
K-nearest neighbor algorithms	0.8176	0.8085	0.8809	0.7911(0.6673,0.8527)

**Figure 2:**
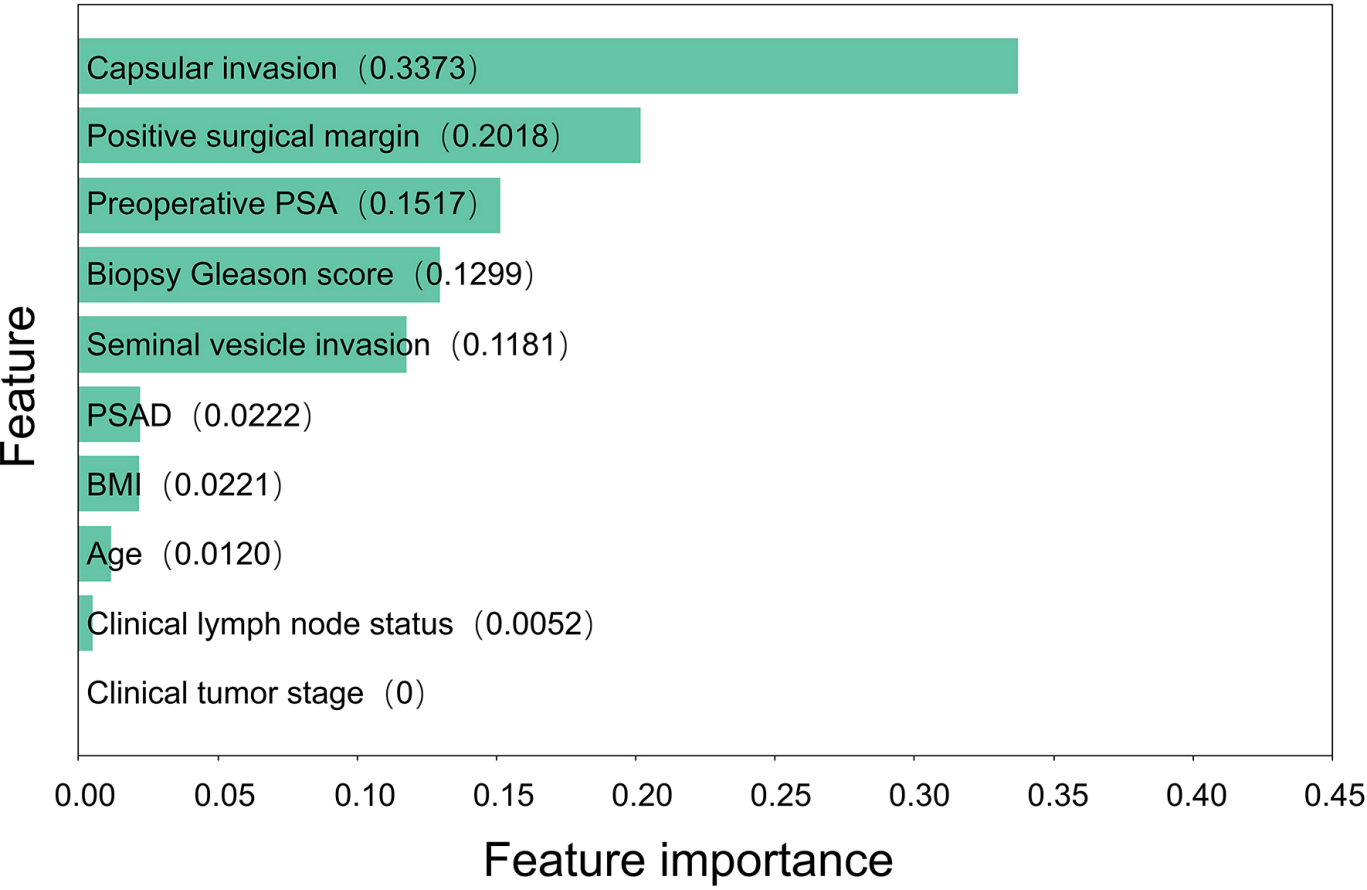
Feature importance of 10 clinical predictors.

Based on the random forest model, we calculated and ranked the importance of factors affecting PSA persistence (**Figure 2**). The results of the importance ranking showed that capsular invasion was the most important factor, followed by a positive surgical margin, preoperative PSA, biopsy Gleason score and seminal vesicle invasion.

## Discussion

Due to its increasing incidence worldwide, prostate cancer has attracted considerable research attention. However, despite this increase in research, radical prostatectomy is still the most important aspect of the surgical treatment of prostate cancer. Patients with nonmetastatic prostate cancer usually have a good prognosis after radical prostatectomy (15–17). However, not all radical prostatectomies can achieve good outcomes. After curative therapy, PSA is the most sensitive and the only validated biomarker of disease recurrence (18, 19). Therefore, PSA is the basis for the follow-up examinations of patients with prostate cancer after RP. Since the half-life of PSA is approximately 3.15 days, serum PSA ≤ 100 ng/ml should be undetectable within 6 weeks after RP (< 0.1 ng/ml) (20). Otherwise, curative treatment is considered to have failed, the patient’s condition should be reevaluated, and a new treatment plan such as salvage radiotherapy should be adopted. The occurrence of PSA persistence indicates the failure of the current treatment regimen, which affects the survival time and quality of life of patients. In the 2024 European Association of Urology (EAU) guidelines for the diagnosis and treatment of prostate cancer, it is noted that the currently recognized postoperative residual PSA primarily originates from three pathways: undetected systemic tumor micro-metastasis prior to surgery, residual localized prostate cancer tissue, and benign prostate tissue remnants. In the first two cases, PSA persistence is of certain value in assessing surgical outcomes and prognosis. Some studies suggest that patients with PSA persistence should initiate early comprehensive treatment plans, such as salvage radiotherapy, endocrine therapy, salvage lymph node dissection, and additional salvage radiotherapy (21). As medical research progresses, many factors, such as higher preoperative PSA, positive surgical margins, and high pathological Gleason score, have been shown to be consistently associated with PSA persistence. However, other risk factors, such as tumor stage and lymph node metastasis, remain controversial (7, 8). Moreover, the adverse consequences of PSA persistence are already relatively clear, but research on its risk factors still needs improvement. In addition, previous research data mostly come from men in Europe and America, lacking studies based on data from Asia. Therefore, if a prediction model for PSA persistence can be established based on data from the Asian region, it would contribute to improving the current understanding of risk factors for PSA persistence, supplement research related to the Asian region, further discussed the application of machine learning in clinical prediction and provide timely postoperative evaluations to guide the selection of treatment plans.

With the continuous development of medical research, the use of big data to establish predictive models is becoming a new hotspot. Predictive models can help us better assess risk, guide clinical management, and reduce unnecessary tests and invasive procedures. Machine learning is a scientific discipline that studies how computers learn from data. It arises at the intersection of statistics, which seeks to learn relationships from data, and computer science, which emphasizes efficient computational algorithms. Through the analysis of an enormous amount of data, reliable statistical models can be built to provide help for medical practice (22). Among these algorithms, random forest, an example of an innovative and highly effective algorithm, is known as one of the best available off-the-shelf classification algorithms. As implied by its name, random forests consist of decision trees. In the present study, we divided patients into two groups, namely, those with and without PSA persistence. Each sample had many characteristics, such as age, preoperative PSA, and BMI. We constructed an ensemble of decision trees, with each individual tree aiming to discriminate between two patient groups based on selected features. At each node of the decision tree, we choose the most effective feature to achieve segmentation. The resulting stochasticity enables each decision tree to independently contribute its vote towards the final classification, serving as a form of regularization. Of course, it is difficult for a single decision tree to be accurate enough, but the summation of hundreds or even more decision tree votes can yield relatively accurate results (13, 23). Statistical experts believe that compared with other common machine learning algorithms, the Random Forest algorithm has certain advantages in terms of its ability to resist overfitting, high accuracy, handling high-dimensional data sets with a large number of features, dealing with missing values, broad applicability, and training speed (24). In a comparative study conducted by the University of Sydney that included 48 studies using machine learning algorithms to construct predictive models, researchers found that the Random Forest algorithm had the highest accuracy (25). In addition to high prediction performance, random forests can also reveal the importance of individual features (26). Due to the advantages of high prediction accuracy and variable importance information for classification, RF has better prediction performance than other machine learning algorithms (25).

Seven commonly used machine learning algorithms were examined herein, and the above ten variables were included to construct the prediction models. When comparing the AUC of the test set of each model, the random forest model had the best performance (0.8011), and the confusion matrix also showed a good accuracy of 0.8156.

Based on this model, we then calculated the importance of each risk factor. The results showed that the five most important risk factors for PSA persistence were capsule invasion, positive surgical margin, preoperative PSA, biopsy Gleason score and seminal vesicle invasion. Prostate cancer with a high preoperative PSA and biopsy Gleason score is more likely to be highly malignant, while capsular invasion and seminal vesicle invasion usually indicate metastasis or even advanced prostate cancer. For patients with highly malignant prostate cancer, the risk of surgical failure should be evaluated more carefully. For patients with a greater risk of metastasis or advanced disease, a more detailed preoperative examination is necessary to evaluate the risk of surgery. Patients with high-risk localized prostate cancer can also undergo lymphadenectomy to reduce the risk of cancer-related death (27). In addition, the incidence of positive surgical margins can be reduced by improvements in surgical methods and preoperative examinations, such as MRI-based diagnostic approaches (28), to reduce the occurrence of PSA persistence. PSAD, BMI, and age were in the second tier of feature importance, suggesting that these commonly used indicators in the assessment of prostate cancer patients may not have a significant impact on PSA persistence. Considering this is a single-center study, the above clinical characteristics may be influenced by regional and hospital factors. In future studies, we can attempt to increase the sample size and collaborate with multiple centers for further investigation.

Our results showed that clinical tumor stage and clinical lymph node status had less influence on the occurrence of PSA persistence. Previous studies have suggested that pathological tumor stage and pathological lymph node status might be risk factors for PSA persistence, but the effect of clinical tumor stage is still unclear (7, 8). This study attempts to utilize more preoperative factors to predict persistent PSA, thereby incorporating clinical staging into the predictive model. However, clinical staging is easily affected by personal experience of the surgeon and imaging modalities, such as magnetic resonance imaging (MRI) and positron emission tomography (PET)-CT, and the performance of examination equipment, so there is a certain difference between clinical staging and pathological status. To improve the credibility of these metrics, it is necessary to provide more standardized training for physicians and invest more in imaging equipment. Therefore, the effect of clinical tumor stage and clinical lymph node status on PSA persistence needs to be further discussed.

Our study also has certain limitations. First, patients with extremely high PSA levels and those who received preoperative adjuvant therapy could not be included in the study since the PSA half-life was approximately 3.15 days. Second, preoperative examination modalities and device performance may have had an impact on our baseline data. Finally, due to the single-center nature of this study, there is a lack of external validation of the prediction effect of the model on populations in different regions. Future improvements in this research field should include multicenter collaboration and more stringent classification criteria.

## Conclusion

The Random Forest algorithm performed excellently in this study and can be used to construct a predictive model for PSA persistence. By incorporating clinical data from the Asian region and exploring the risk factors for PSA persistence, this study contributes to the existing research and aids clinicians in assessing the risk of PSA persistence occurrence, enabling timely treatment planning and improving patient prognosis.

## Data Availability

The raw data supporting the conclusions of this article will be made available by the authors, without undue reservation.
